# The results of Muller Muscle Conjunctival Resection versus Levator Advancement for mild to moderate ptosis

**DOI:** 10.22336/rjo.2023.23

**Published:** 2023

**Authors:** Nese Arslan, Alperen Bahar, Mutlu Acar, Mustafa Kosker, Naciye Kabatas, Canan Gurdal

**Affiliations:** * Department of Ophtalmology, Diskapi Yıldırım Beyazıt Training and Research Hospital, Ankara, Turkey; ** Dunya Goz Hospital Groups, Ankara, Turkey

**Keywords:** Muller muscle conjunctiva resection, External levator advancement, eyelid ptosis

## Abstract

**Purpose:** To compare the surgical outcomes of Muller muscle conjunctival resection (MMCR) and levator advancement (LA) in patients with mild to moderate blepharoptosis.

**Methods:** A retrospective review of patients who underwent surgery for mild to moderate ptosis between 2015 and 2020 was performed. The degree of ptosis was graded based on the amount of upper eyelid drooping: mild ≤ 2 mm and moderate < 4 mm. Surgical success was defined as post-operative marginal reflex distance 1 (MRD1) ≥ 4.0 and ≤ 5.0 mm, and a satisfactory eyelid contour.

**Results:** A total of 82 eyes of 65 patients underwent ptosis repair surgeries. MMCR was performed in 48 eyes and LA in 34 eyes. Under-correction was detected in 8.3% and 11.8% of the patients in MMCR group and LA group respectively. There was no patient with over-correction in the MMCR group postoperatively, 3 patients in the LA group had over-correction (0% vs. 8.8% respectively). The success rate in our study was found to be 91.7% in the MMCR group and 72.2% in the LA group.

**Conclusions:** The MMCR and LA procedures are effective approaches in treating patients with mild to moderate eyelid ptosis in our population. Each procedure had its superiority in selected groups of patients. However, the complication rate and duration of surgery were found to be lower in MMCR group.

**Abbreviations: **LA = Levator Advancement, LF = Levator Function, MMCR = Muller Muscle Conjunctival Resection, MRD 1 = Marginal Reflex Distance

## Introduction

Muller muscle-conjunctival resection (MMCR) and external levator advancement (LA) are two favored techniques for ptosis repair [**1**-**3**]. The effectiveness of both procedures in mild to moderate ptosis has been previously shown in different populations [**3**-**6**]. The MMCR was first introduced by Putterman and Urist [**1**] and has been widely performed in mild ptosis, which improves with the instillation of phenylephrine [**7**]. But, the effect of MMCR on correcting mild to moderate ptosis is not clear [**4**,**8**,**9**]. On the other hand, LA is among the most commonly performed external repairs used today [**10**,**11**]. The aim of our study was to evaluate the surgical outcomes of both MMCR and LA in the treatment of mild to moderate ptosis in cases with isolated congenital and aponeurotic blepharoptosis.

## Materials and methods

This study followed the guidelines of the Declaration of Helsinki and was approved by the ethical committee of Diskapi Yildirim Beyazit Training and Research Hospital, University of Health Sciences (16.03.2020/ 84/ 13). 

In this retrospective study, the medical records of 82 surgically treated eyes of 65 patients with mild-moderate ptosis were reviewed between 2015-2020 at Diskapi Yildirim Beyazit Training and Research Hospital. These patients were grouped according to the surgical procedure as MMCR group and LA group. The data collected included demographics, etiology, pre-operative marginal reflex distance 1 (MRD1), levator function (LF) and surgical outcomes. Surgical outcomes were evaluated as primary and secondary outcomes. Primary outcomes included MRD1 and LF measured at 1st month and 6th months postoperatively, while postoperative complications were evaluated as secondary outcomes, including under-correction, over-correction, and lid contour abnormalities. The surgical duration was compared between groups. This period, starting with local anesthesia and ending with saturation, was obtained from the surgical records based on the operation room timer.

The amount of ptosis was assessed according to the normal MRD1 measurement (4.0-5.0 mm) and graded based on the amount of upper eyelid drooping: mild ≤ 2 mm and moderate < 4 mm [**12**,**13**]. 

The LF is defined as the distance between the excursion of upper lid margin from full downgaze to full up gaze with frontalis muscle function neutralized [**14**-**16**]. In our study, LF ≥ 10 mm was considered as good LF. The phenylephrine test was performed by applying 1 drop of 2.5% phenylephrine solution to the eye. Response to phenylephrine test was defined as the change in MRD1 (at least 1 mm elevation) [**17**]. MRD1 measurements performed before and 10-15 min. after the installation of topical phenylephrine were recorded. MMCR was performed in patients who showed improvement in ptosis with phenylephrine eye drops. Eight millimeters of Müller muscle were resected for 1 millimeter of ptosis correction with a maximum of 10 mm for ptosis over 2 mm. LA procedure was considered when there was no response to phenylephrine. Patients with previous eyelid trauma or surgery, significant corneal disease, and advanced glaucoma were excluded. Only patients with isolated congenital and aponeurotic ptosis were included. In cases of asymmetric bilateral ptosis, the amount of resection was planned according to the amount of ptosis and the LF. 

Surgical success was defined as post-operative MRD1 ≥ 4.0 and ≤ 5.0 mm, inter-eyelid asymmetry within 1 mm of normal, and a satisfactory eyelid contour [**18**-**20**].

**Statistical Analysis**

Statistical analysis was performed using IBM SPSS Statistics for Windows v.21.0 (IBM Corp., Armonk, NY, USA). Descriptive statistics are presented as mean ± SD. The chi-square test was used for categorical variables. Parametric variables were compared between groups via analysis of variance and t-test. Nonparametric data were compared using the Kruskal-Wallis and Mann-Whitney U tests, as appropriate. The level of statistical significance was set at p < 0.05.

## Results

A total of 82 eyes of 65 patients underwent ptosis repair surgeries. Seventeen cases underwent bilateral ptosis surgery. MMCR was performed in 48 eyes and LA in 34 eyes. The difference in the mean age, gender distribution, and etiology were not significant between groups (**Table 1**). The mean age of patients with congenital ptosis was significantly lower than the mean age of patients with aponeurotic ptosis (p=0,001). Although the mean age of cases with successful surgical results was higher (49,9 ± 16,8 years), it was not found statistically significant because of the low number of cases with surgical failure (39,5 ± 18,4 years) (p=0,07). MRD1 measurements were significantly lower in the LA group than those in the MMCR group preoperatively. However, there was no difference in MRD1 measurements at 1st month and 6th months postoperatively (**Table 2**). 

**Table 1:** Demographic characteristics of the study

	MMCR	LA	p
Etiology			
Congenital	12 (25%)	12 (35.3%)	0.54
Aponeurotic	36 (75%)	22 (64.7%)	0.09
Age			
Total	48.9 ± 16	47.7 ± 18.7	0.86
Congenital	27.3 ± 15.6	29.8 ± 13.5	0.56
Aponeurotic	54.2 ± 13.2	57.5 ± 13.1	0.36
Successful cases	50,7 ± 15,6	48,4 ± 19	0,6
Congenital	33,3 ± 15,6	25,4 ± 7,3	0,2
Aponeurotic	55,9 ± 11,3	58,0 ± 13,0	0,5
Gender			
Female	29 (60.4%)	17 (50%)	0.37
Congenital	9 (18.7%)	2 (5.9%)	0.012
Aponeurotic	20 (41.7%)	15 (44.1%)	0.23
Male	19 (39.6%)	17 (50%)	0.43
Congenital	3 (6.3%)	10 (29.4%)	0.014
Aponeurotic	16 (33.3%)	7 (20.6%)	0.47

**Table 2:** Primary surgical outcomes of the study

	MMCR	LA	p
**MDR1** (mm)			
Preoperative	2.3 ± 0.46	2.1 ± 0.1	0.001
Congenital	2.5 ± 0.52	2.0 ± 0.1	0.001
Aponeurotic	2.2 ± 0.4	2.0 ± 0.1	0.003
Postoperative 1. Month	4.1 ± 0.2	4.3 ± 0.9	0.24
Congenital	4.0 ± 0.9	3.7 ± 0.6	0.33
Aponeurotic	4.1 ± 0.4	4.1 ± 0.6	0.72
Postoperative 6. Month	4.1 ± 0.6	4.0 ± 0.8	0.22
Congenital	4.0 ± 0.7	3.8 ± 0.5	0.34
Aponeurotic	4.2 ± 0.5	4.2 ± 0.7	0.26
Absolute Change	1.8 ± 0.4	1.9 ± 0.3	0.15
**LF ** (mm)	MMCR	LA	p
Preoperative	13.8 ± 2.4	10.9 ± 1.0	0.001
Congenital	13.33 ± 2.4	10.8 ± 0.8	0.004
Aponeurotic	13.9 ± 2.4	11.0 ± 1.0	0.001
Postoperative 1. Month	13.9 ± 2.3	11.1 ± 1.03	0.001
Congenital	13.34 ± 2.3	10.9 ± 0.9	0.005
Aponeurotic	13.9 ± 2.4	11.22 ± 1.2	0.001
Final visit	13.9 ± 2.3	11.1 ± 1.03	0.001
Congenital	13.5 ± 2.4	10.8 ± 0.9	0.004
Aponeurotic	13.9 ± 2.4	11.24 ± 1.04	0.001
p	0.34	0.46	

**Fig. 1:**
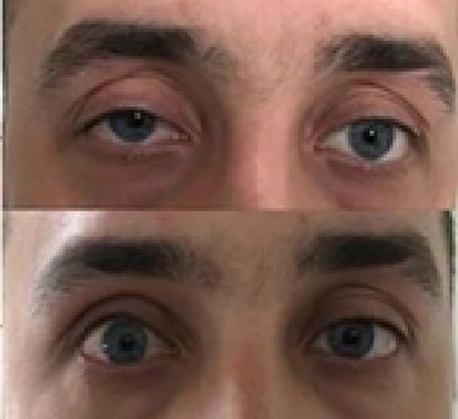
Over-correction in the right eye after levator advancement surgery

Four patients in the MMCR group and four patients in the LA group underwent under-correction (8.3% vs. 11.8%). Of 4 patients who underwent under-correction in the LA group, 3 were re-operated with levator resection. In the MMCR group, 2 of the 4 under-corrected patients were re-operated with levator resection. One patient in the LA group and 2 patients in the MMCR group did not accept a second surgery. No patient with over-correction was registered in the MMCR group, while 3 patients in the LA group underwent over-correction (0% vs. 8.8%) (**Fig. 1**). In the LA group, 2 of them were re-operated with suture recession, and one patient was informed to apply massage to the upper eyelid postoperatively. Lid contour abnormalities developed only in 1 patient in the LA group (2,3%). The success rate was higher in the MMCR group (91.7%) than in the LA group (72.2%). The mean surgical duration was 20,4 ± 3,9 min. (15-30) in the MMCR group and 44,9 ± 8,6 min. (30-70) in the LA group. The difference between the groups was statistically significant (p=≤0,001) (**Table 3**).

**Table 3:** Secondary surgical outcome

Complication	MMCR	LA	p
Under-correction	4 (8.3%)	4 (11.8%)	0.23
Congenital	2 (16.7%)	3 (25%)	
Aponeurotic	2 (5.6%)	1 (4.6%)	
Over-correction	0	3 (8.8%)	0.067
Congenital		1 (8.3%)	
Aponeurotic		2 (9.1%)	
Lid counter abnormalities		1 (2.9%)	0.4
Congenital			
Aponeurotic		1 (4.6%)	
Success rate	91.7% (44/ 48)	72.2% (26/ 34)	0.012
Congenital	83.3% (10/ 12)	66.7% (8/ 12)	
Aponeurotic	94.4% (34/ 36)	81.8% (18/ 22)	
**Surgical duration** (min.)	20,4 ± 3,9	44,9 ± 8,6	≤ 0.001

## Discussion

Since its first proposal by Putterman [**1**], MMCR has been performed using different nomograms as an efficient and safe procedure for the correction of mild ptosis, because of the relatively predictable operative results, minimal damage to the tarsal plate, and the lacrimal glands, short operation time, good postoperative eyelid contour, and the fact that no skin incision is needed compared with past surgical methods [**4**]. On the other hand, the main advantages of LA include suitability for all degrees of ptosis, the ability to make intraoperative adjustments, and preservation of the conjunctiva. Although it is possible to remove excess skin in MMCR, the external approach allows the removal of excessive skin and fat from the same incision if needed. Disadvantages of the LA include a steeper learning curve, longer surgical times compared to MMCR, less predictability, and increased risk of complications such as over/ under correction and abnormal lid contour [**21**-**24**]. MMCR efficacy on moderate and severe ptosis with or without respect to a phenylephrine response has been a subject of debate. A variety of algorithms relating to the amount of tissue resected has been experienced. Surgical success rates in the literature ranged from 70-95% for LA [**25**-**27**] and 80-100% for MMCR [**28**,**29**]. 

In this study, both procedures showed effective surgical outcomes, but the success rate was higher in MMCR (MMCR: 91,7%, LA: 72.2%), in accordance with literature. In addition, we assessed the success rate between groups according to the etiology and we found that the success rate was lower in both procedures in cases with congenital ptosis (MMCR: 83,3%; LA: 66,7%) than in those with aponeurotic ptosis (MMCR: 94,4%; LA: 81,8%). To the best of our knowledge, this is the first study to compare success rates between two different procedures according to the etiology. Thomas et al. [**3**] achieved a success rate of 96,5% in the MMCR group and 79,3% in the LA group. Whereas Sweeny et al. [**30**] have recently reported a success rate of 90.9% in MMCR group and 85% in LA in patients with mild to moderate ptosis. They also demonstrated that MMCR was an effective approach in treating patients with severe ptosis, and it might have superiority to external LA [**30**]. 

In this study, although there was no difference regarding the mean age between the MMCR and LA groups, the mean age of cases with congenital ptosis was significantly lower than the mean age of cases with aponeurotic ptosis (p=0,001). The mean age of cases with successful surgical results was higher, but it was not found statistically significant (p=0,07). To the best of our knowledge, this was the first study to evaluate the effect of age on success according to the surgical procedure and etiology. As we evaluated the gender distribution in our study, the female number was higher in the MMCR group, but still, the difference was not statistically significant. The female-to-male ratio in our study was 1,5:1 in the MMCR group and 1:1 in the LA group. Female to male ratio was 19:1 and 15:5 in Saonanon et al.’s study [**5**]. Ptosis has a wide spectrum of etiology including myogenic, neurogenic, aponeurotic, mechanical, or traumatic causes [**31**,**32**]. In this study, we included only isolated congenital and aponeurotic ptosis, in which the difference in etiology distributions between groups was not statistically significant. Most of the patients in our study had aponeurotic blepharoptosis, in accordance with the data in literature [**4**]. 

Although the preoperative values of MRD1 were significantly different between the two groups, there was no substantial difference in the final postoperative MRD1 in both groups. Besides the absolute change in MRD1, 1.8 vs. 1.9 mm respectively, p=0.25 was not significant. Thomas et al. reported that there was no significant difference in the final postoperative MRD1 in either group. Yet, in their study, the absolute change in MRD1 was significantly inconsistent in the MMCR group (MMCR: 1,76 mm and LA: 2,93 mm) [**3**]. They attributed this result to higher preoperative MRD1 levels in the MMCR group. Saonanon et al. [**5**] demonstrated in their study that the postoperative MRD1 was 3,2 ± 1,0 mm in the MMCR group and 3,0 ± 1,0 mm in the LA group and the mean change in the MRD1 was not statistically significant (p=0,978). 

In terms of postoperative complications, Abrishami et al. [**33**] reported under-correction in 19,1% of the patients and over-correction in 2,2% of the patients who underwent levator resection surgery. Simon GJ et al. [**6**] found about 26% under-correction in the LA and 4% in the MMCR groups, whereas over-correction and lid retraction rate were reported as 1,4% in all procedures. Thomas et al. [**3**] observed higher complication rates in the LA group. These rates were summarized as over-correction (13%) and lid contour abnormality (20%), observed only in the LA group (p<0,05). Under-correction was reported in both groups (MMCR: 10%, LA: 7%; p=0,61). Our results showed an approximately equal risk of under-correction in both groups (MMCR: 8,3%, LA: 11,8%; p=0,7), whereas over-correction (MMCR: 0%, LA: 8,8%; p=0.07) and lid contour abnormality (MMCR 0%, LA 3%; p=0,4) rates were higher in the LA group, but they were not considered statistically significant.

In literature, different results have been reported on surgical duration [**5**,**34**]. Liao SL et al. reported that the operation period (MMCR: 15 min., LA: 30-45 min.) and the recovery time were shorter in MMCR in comparison to LA. They also reported that the MMCR procedure is simple, case-independent, and the results are predictable, with no compromise of the eyelid crease contour [**35**]. In our study, the MMCR group showed a shorter surgical duration compared with the LA group, in accordance with literature (MMCR: 20,4 ± 3,9 min.; LA: 44,9 ± 8,6 min.; p=0,001). 

Limitations of this study, including the selection bias, were that it was a retrospective uncontrolled study of two different groups with better LF, preoperative MRD1 in the MMCR group. Lower rate of cases with congenital ptosis in the MMCR group (25%) than the LA group (35,3%) could have skewed our results to make the MMCR group show better results than the LA group. Both shorter surgical procedures and lower probability of complications might have played an important role in the higher rate of success in the MMCR procedure. Lack of short-term evaluation of the healing process, scar formation and patient satisfaction were other limitations of this study. 

## Conclusion

In conclusion, our surgical results showed that both the MMCR and LA procedures are effective in the correction of mild to moderate upper eyelid ptosis in our population, with more predictable results after MMCR procedure. Even though the procedures were quite different in terms of surgical process and indications, both showed high success rates in ptosis correction. The MMCR group showed lower rates of complications and shorter duration of the surgical process. Based on these findings, we recommend the consideration of performing MMCR in patients with mild to moderate ptosis, who show response to phenylephrine.

**Conflict of Interest statement**

The authors declare there is no conflict of interest or relation.

**Informed Consent and Human and Animal Rights statement**

Informed consent has been obtained from all individuals included in this study.

**Authorization for the use of human subjects**

Ethical approval: The research related to human use complies with all the relevant national regulations, institutional policies, is in accordance with the tenets of the Helsinki Declaration, and has been approved by ethical committee of Diskapi Yildirim Beyazit Training and Research Hospital, University of Health Sciences (16.03.2020/84/13), Ankara, Turkey.

**Acknowledgements**

None.

**Sources of Funding**

No financial support was received for this manuscript.

**Disclosures**

None.
